# Political Party Affiliation and COVID-19 Vaccination Status in Japan, 2022

**DOI:** 10.7759/cureus.66035

**Published:** 2024-08-02

**Authors:** Tomohiko Ukai, Ayu Kasamatsu, Takahiro Tabuchi

**Affiliations:** 1 Epidemiology and Clinical Research, Research Institute of Tuberculosis, Kiyose, JPN; 2 Division of Molecular Epidemiology, Jikei University School of Medicine, Tokyo, JPN; 3 Cancer Control Center, Osaka International Cancer Institute, Osaka, JPN

**Keywords:** cross sectional studies, cohort, covid-19 vaccine, vaccine science and policy, vaccine hesitancy, vaccination policy, covid-19

## Abstract

Introduction: An association has been reported between political affiliations and vaccination worldwide. In Japan, a significant proportion of the population are non-partisans, and major political parties advocate COVID-19 vaccination. The association between supporting political parties and COVID-19 vaccination coverage in Japan remains unclear. This study aims to investigate the relationship between political party affiliation and COVID-19 vaccination status in Japan.

Methods: This study utilized data from large-scale nationwide internet surveys conducted in Japan in 2022, with a sample size of 21,162 participants. The surveys collected information on participants' COVID-19 vaccination status and political party affiliation. The political parties included in the analysis were the Liberal Democratic Party, the Constitutional Democratic Party, the Komeito, the Japanese Communist Party, the Japan Innovation Party (Nippon Ishin no Kai), and the Reiwa Shinsengumi, as well as non-partisans. Logistic regression analysis was performed to examine the relationship between political partisanship and COVID-19 vaccine status. The analysis controlled for potential confounding variables such as age, gender, socioeconomic status, and geographic location.

Results: The odds of being unvaccinated were lower for supporters of large political groups (e.g. Liberal Democratic Party {OR 0.6; 95% CI, 0.5-0.7}), while higher for small political groups (e.g. Reiwa Shinsengumi {OR 2.6; 95% CI, 1.9-3.6}), in comparison with non-partisan.

Conclusion: Political affiliation may be associated with vaccination disparities in Japan. Supporters of minor parties were more likely to be unvaccinated than those of the larger parties. However, this study has several limitations, including self-reporting bias and selection bias due to the Internet survey methodology.

## Introduction

It is assumed that there is an association between vaccination and political affiliations. In the United States, the anti-vaccine stance of the former Republican president led to low COVID-19 vaccination coverage among Republicans [1]. Similarly, right-wing parties have lower vaccination coverage than their left-wing counterparts [2]. However, this disparity was less pronounced when cross-partisan consensus against the COVID-19 vaccine was achieved [3]. In Japan, one of the countries with the highest COVID-19 vaccination coverage, the relationship between vaccination and political support is poorly understood. A sizable Japanese population has been identified as non-partisan, and the ruling Liberal Democratic Party (LDP) and the major opposition parties, such as the Japan Innovation Party (Nippon Ishin no Kai) and Constitutional Democratic Party (CDP), advocate for COVID-19 vaccination. This study aimed to identify the association between supporting political parties and COVID-19 vaccination coverage in Japan.

## Materials and methods

This cross-sectional study was based on two nationwide online surveys. The Japan Society and New Tobacco Internet Survey (JASTIS) 2022 was conducted in February 2022, and the Japan COVID-19 and Society Internet Survey (JACSIS) 2022 was conducted in September and October 2022. The JASTIS evaluated the status of new tobacco products and sociodemographic factors among the Japanese general population. The JACSIS evaluated the health conditions and social determinants of the COVID-19 pandemic in Japan. Both surveys were administered through internet questionnaires. Both surveys shared the same randomly sampled respondents aged 16 to 82 years who were invited to participate from a large survey panel managed by a major nationwide internet research agency, Rakuten Insight, Inc., Tokyo, Japan. The survey was terminated once the target number of respondents was reached for each category (sex, age, and prefecture). The details of the surveys have been described elsewhere [4,5]. Of 33,000 individuals who responded to JASTIS 2022 and 32,000 who responded to JACSIS 2022, we included 27,636 who responded to both surveys. To validate the quality of the data, we excluded responses with discrepancies or artificial, unnatural responses [6]. We also excluded minors who were not eligible to vote and those who selected the option “not be eligible for vaccination due to allergies or other health conditions” as their answer to the question on vaccination status. This study was conducted in accordance with the guidelines of the Declaration of Helsinki, and all procedures involving the participants were approved by the Ethics Committee of the Osaka International Cancer Institute (no. 20084).

Political party affiliation

In JASTIS 2022, respondents were queried on their political party affiliation by asking which party they supported. The options presented were nine main political parties in Japan, other parties, and no party affiliation [7].

Vaccination status

In Japan, the primary series of COVID-19 vaccinations began in February 2021, with the government fully funding vaccination for citizens under the Immunization Act. JACSIS 2022 surveyed participants on their COVID-19 vaccination status as of August 2022. Participants who had not received any dose or had received only once were categorized as unvaccinated. Participants who received at least two doses were categorized as being vaccinated.

Statistical analysis

The proportion of respondents by political parties was presented via other political party support polls conducted online or the phone in February 2022 [8]. In addition, the characteristics of respondents by the political parties in our study were described by number and proportion. We explored the association between political party affiliation and being unvaccinated by 2022. Univariable odds ratios (ORs) and 95% confidence intervals (CIs) of political party affiliation for unvaccinated were calculated using logistic regression analysis. In multivariate analysis, the model was adjusted for age (category), sex (binary), final education (category), annual household income (category), employment status (category), living area (category), and history of COVID-19 infection (binary) [9,10]. We utilized inverse probability weighting based on propensity scores to derive estimates generalizable to the Japanese population from the sample of respondents for the proportion of unvaccinated supporters and the odds ratios for being unvaccinated [6]. All analyses were performed using SAS 9.4 (SAS Institute, Cary, NC, USA).

## Results

Of the 27,636 respondents who completed the two questionnaires, 6240 with discrepancies or artificial/unnatural responses, 6 minors, and 235 with exemptions were excluded from the analysis, resulting in a final sample of 21,155 respondents for analysis. Among the political parties, the LDP, Japan Innovation Party, and CDP had the most supporters, with 4,409 (20.8%), 1,794 (8.5%), and 1,066 (5.0%), respectively, and the largest group was the non-partisan group (12,087, 57.1%). Table 1 shows the basic demographic characteristics of participants by political affiliation. The proportion of those aged 61 years and older was the highest among CDP supporters (64.8%) and tended to be lower among minor party supporters (e.g., NHK party, 24.3%) and non-partisan individuals (26.9%).

**Table 1:** Basic characteristics of participants by partisanship. JPY: Japanese yen.

		Liberal Democratic Party (n=4409)	Komeito (n=417)	Constitutional Democratic Party of Japan (n=1065)	Democratic Party for the People (n=308)	Social Democratic Party (n=111)	Japan Innovation Party (Nippon Ishin no Kai) (n=1794)	Japanese Communist Party (n=504)	Reiwa Shinsengumi (n=212)	NHK Party (n=137)	Other (n=111)	Non-partisan (n=12087)
Characteristic		n (%)	n (%)	n (%)	n (%)	n (%)	n (%)	n (%)	n (%)	n (%)	n (%)	n (%)
Age (years)	18–20	53(1.2)	11(2.6)	14(1.3)	15(4.9)	2(1.8)	18(1.0)	5(1.0)	5(2.4)	3(2.2)	3(2.7)	251(2.1)
	21–40	1036(23.5)	97(23.3)	134(12.6)	137(44.5)	26(23.4)	394(22.0)	60(11.9)	61(28.8)	64(46.7)	42(37.8)	4042(33.4)
	41–60	1521(34.5)	137(32.9)	227(21.3)	86(27.9)	19(17.1)	592(33.0)	136(27.0)	77(36.3)	47(34.3)	39(35.1)	4549(37.6)
	61–80	1767(40.1)	170(40.8)	669(62.8)	68(22.1)	64(57.7)	775(43.2)	301(59.7)	68(32.1)	23(16.8)	26(23.4)	3202(26.5)
	81–	32(0.7)	2(0.5)	21(2.0)	2(0.6)	0(0.0)	15(0.8)	2(0.4)	1(0.5)	0(0.0)	1(0.9)	43(0.4)
Sex	Male	2727(61.9)	190(45.6)	639(60.0)	216(70.1)	62(55.9)	1052(58.6)	258(51.2)	132(62.3)	104(75.9)	72(64.9)	5114(42.3)
	Female	1682(38.1)	227(54.4)	426(40.0)	92(29.9)	49(44.1)	742(41.4)	246(48.8)	80(37.7)	33(24.1)	39(35.1)	6973(57.7)
Final education	High school	1193(27.1)	177(42.4)	333(31.3)	81(26.3)	33(29.7)	467(26.0)	125(24.8)	66(31.1)	40(29.2)	37(33.3)	3489(28.9)
	Undergraduate	786(17.8)	84(20.1)	165(15.5)	50(16.2)	19(17.1)	376(21.0)	91(18.1)	39(18.4)	28(20.4)	17(15.3)	2775(23.0)
	University	2118(48.0)	140(33.6)	500(46.9)	156(50.6)	51(45.9)	849(47.3)	259(51.4)	93(43.9)	61(44.5)	47(42.3)	5184(42.9)
	Postgraduate	294(6.7)	14(3.4)	61(5.7)	19(6.2)	7(6.3)	96(5.4)	28(5.6)	13(6.1)	6(4.4)	6(5.4)	543(4.5)
	Other	18(0.4)	2(0.5)	6(0.6)	2(0.6)	1(0.9)	6(0.3)	1(0.2)	1(0.5)	2(1.5)	4(3.6)	96(0.8)
Employment status	Executive	224(5.1)	10(2.4)	30(2.8)	12(3.9)	7(6.3)	83(4.6)	12(2.4)	18(8.5)	6(4.4)	7(6.3)	352(2.9)
	Office work	1660(37.7)	130(31.2)	246(23.1)	138(44.8)	25(22.5)	641(35.7)	109(21.6)	66(31.1)	60(43.8)	41(36.9)	4449(36.8)
	Self-employment	319(7.2)	34(8.2)	72(6.8)	23(7.5)	9(8.1)	140(7.8)	37(7.3)	26(12.3)	19(13.9)	11(9.9)	780(6.5)
	Part-time	581(13.2)	96(23.0)	157(14.7)	49(15.9)	19(17.1)	280(15.6)	101(20.0)	35(16.5)	17(12.4)	22(19.8)	2267(18.8)
	Housework	665(15.1)	66(15.8)	181(17.0)	24(7.8)	20(18.0)	289(16.1)	77(15.3)	26(12.3)	8(5.8)	6(5.4)	1947(16.1)
	Retired	251(5.7)	12(2.9)	138(13.0)	11(3.6)	8(7.2)	110(6.1)	47(9.3)	8(3.8)	2(1.5)	5(4.5)	436(3.6)
	Student	122(2.8)	13(3.1)	22(2.1)	23(7.5)	2(1.8)	33(1.8)	9(1.8)	8(3.8)	8(5.8)	4(3.6)	532(4.4)
	Not employed	587(13.3)	56(13.4)	219(20.6)	28(9.1)	21(18.9)	218(12.2)	112(22.2)	25(11.8)	17(12.4)	15(13.5)	1324(11.0)
Annual household income (JPY)	0	28(0.6)	5(1.2)	10(0.9)	3(1.0)	2(1.8)	6(0.3)	6(1.2)	0(0.0)	1(0.7)	1(0.9)	154(1.3)
	1–5,000,000	1664(37.7)	181(43.4)	515(48.4)	121(39.3)	56(50.5)	729(40.6)	264(52.4)	107(50.5)	61(44.5)	57(51.4)	4511(37.3)
	5,000,001–10,000,000	1505(34.1)	119(28.5)	292(27.4)	121(39.3)	32(28.8)	609(33.9)	126(25.0)	62(29.2)	41(29.9)	22(19.8)	3371(27.9)
	10,000,001–20,000,00	567(12.9)	31(7.4)	85(8.0)	23(7.5)	3(2.7)	184(10.3)	35(6.9)	21(9.9)	9(6.6)	11(9.9)	921(7.6)
	20,000,001–	79(1.8)	2(0.5)	10(0.9)	3(1.0)	2(1.8)	24(1.3)	5(1.0)	1(0.5)	2(1.5)	4(3.6)	110(0.9)
	Do not want to answer	317(7.2)	41(9.8)	96(9.0)	22(7.1)	7(6.3)	140(7.8)	42(8.3)	7(3.3)	9(6.6)	7(6.3)	1374(11.4)
	Do not know	249(5.6)	38(9.1)	57(5.4)	15(4.9)	9(8.1)	102(5.7)	26(5.2)	14(6.6)	14(10.2)	9(8.1)	1646(13.6)
History of COVID-19	No	4133(93.7)	387(92.8)	1030(96.7)	285(92.5)	91(82.0)	1664(92.8)	482(95.6)	194(91.5)	122(89.1)	101(91.0)	11327(93.7)
	Yes	276(6.3)	30(7.2)	35(3.3)	23(7.5)	20(18.0)	130(7.2)	22(4.4)	18(8.5)	15(10.9)	10(9.0)	760(6.3)

Of the participants, 89.3% (18,889/21,155) received at least two doses of the COVID-19 vaccine. The weighted proportions of unvaccinated individuals among supporters of the LDP, the Japan Innovation Party, and the CDP were 6.3%, 6.8%, and 4.3%, respectively. Conversely, the weighted proportion of unvaccinated individuals was higher among supporters of minor political parties, such as Reiwa Shinsengumi (26.0%), NHK Party (22.6%), and other parties (35.1%). The proportion of being unvaccinated among non-partisan individuals was 14.6%. In multivariate analysis, the odds of being unvaccinated were lower for large political groups (LDP {OR 0.6; 95% CI, 0.5-0.7}, Japan Innovation Party {OR 0.6; 95% CI, 0.6-0.9}, CDP {OR 0.5; 95% CI, 0.4-0.7}), while higher for supporters of small political groups (Reiwa Shinsengumi {OR 2.6; 95% CI, 1.9-3.6}, NHK Party {OR 2.1; 95% CI, 1.4-3.2}, and other parties {OR 3.4; 95% CI, 2.2-5.1}) in reference to non-partisan individuals (Table 2).

**Table 2:** Partisanship and odds of being unvaccinated (N=21,155). CI, confidence interval. a Adjusted for age, sex, final education, annual household income, employment status, living area, and history of COVID-19. b We calculated estimates that are representative of the general population from the sample of respondents, adjusted using inverse probability weighting. NHK: Nippon Hoso Kyokai (Japan Broadcasting Corporation).

	Number of supporters, n	Unvaccinated supporters, n	Unvaccinated supporters, % (unweighted)	Unvaccinated supporters, % (weighted^b^)	Odds-ratio (univariable) (95% CI) (weighted^b^)	Odds-ratio (multivariable^a^) (95% CI) (weighted^b^)
Political affiliation						
Non-partisan	12087	1507	12.5	14.6	ref	ref
Liberal Democratic Party	4409	308	7.0	6.3	0.5(0.5-0.6)	0.6(0.5-0.7)
Komeito	417	35	8.4	6.8	0.6(0.5-0.9)	0.7(0.5-1.0)
Constitutional Democratic Party of Japan	1065	50	4.7	4.3	0.3(0.3-0.5)	0.5(0.4-0.7)
Democratic Party for the People	308	27	8.8	10.9	0.7(0.5-1.0)	0.6(0.4-0.9)
Social Democratic Party	111	18	16.2	17.2	1.4(0.8-2.3)	1.6(1.0-2.8)
Japan Innovation Party (Nippon Ishin no Kai)	1794	147	8.2	6.8	0.6(0.5-0.7)	0.7(0.6-0.9)
Japanese Communist Party	504	43	8.5	11.4	0.7(0.5-0.9)	0.9(0.6-1.2)
Reiwa Shinsengumi	212	56	26.4	26.0	2.5(1.8-3.4)	2.6(1.9-3.6)
NHK Party	137	37	27.0	22.6	2.6(1.8-3.8)	2.1(1.4-3.2)
Other	111	38	34.2	35.1	3.7(2.5-5.4)	3.4(2.2-5.1)

## Discussion

COVID-19 has led to numerous global fatalities and significant political and social consequences. Vaccination stands as the primary strategy to mitigate severe forms of infections and deaths [11]. However, vaccine hesitancy, recognized as a top threat by the World Health Organization [12], poses a serious public health challenge. Thus, a better understanding of the vaccination status distribution is essential to combat this issue [13]. In this study, differences in the COVID-19 vaccination status among supporters of different political parties in Japan were noted. To the best of our knowledge, this is the first study to explore the association between supporting political parties and vaccination status in Japan.

The association between political affiliation and vaccine uptake has been reported in the literature from other countries. Right-wing parties have been shown to exhibit lower vaccine coverage compared with left-wing parties [1,2]. This may primarily be attributed to the emphasis right-wing parties place on individual liberty, their tendency to avoid perceived manufactured risks, and their propensity to endorse conspiracy theories [2]. In Japan, however, major political parties, including right-wing and left-wing parties, have demonstrated high vaccination coverage. In contrast, minor political parties and those without partisan affiliation have exhibited low coverage. This trend may be attributed in part to the official endorsement of COVID-19 vaccination by major political parties, similar to the policies of countries such as the United Kingdom and Canada, where both ruling right-wing parties and opposing left-wing parties officially advocate vaccination [3,10]. Japan’s overall high vaccination coverage may reflect the high rates among major parties with a substantial proportion of supporters.

Minor political parties tended to have higher odds of being unvaccinated. Similar trends were observed in France and Australia [14,15]. The authors suggest this may be due to differing perceptions towards those in governance, though the underlying reasons are not fully understood. Other factors, such as restricted information access and lower socio-economic status may also contribute [16]. Notably, non-partisans, making up 67.7% of the unvaccinated, exhibit greater vaccine hesitancy than those affiliated with major parties. Achieving LDP-level vaccination rates among non-partisans could result in 6.1 million additional immunizations, considering Japan's population and the proportion of unvaccinated supporters. The potential social isolation of non-partisans might correlate with their vaccination status [17]. Although the underlying personal and structural factors contributing to vaccination differences based on political affiliation are beyond the scope of our analysis, understanding these factors is important for developing targeted strategies to overcome vaccination barriers.

In this study, we found that individuals of older age and higher income tended to support major political parties and also had higher vaccination uptake rates. This result suggests that socioeconomic factors such as age and income influence vaccination behavior. Older individuals may have a higher awareness of health risks and a better understanding of the importance of vaccinations. Additionally, higher-income individuals likely have easier access to health information and more opportunities to get vaccinated. Furthermore, supporters of major political parties might have a stronger trust in public health policies and vaccination programs. These combined factors likely contribute to the higher vaccination rates observed among older and higher-income individuals who support major political parties.

Limitations of the study

This study has several limitations. First, the self-reported nature of the questionnaire survey could introduce recall bias or social desirability bias into the reported vaccination status and political party preference. Second, the study’s observational design may have resulted in residual confounding, such as underlying conditions or access to information, despite adjusting for various factors. Third, non-internet users were not included in the survey, which could have resulted in a selection bias. However, individual internet use in Japan is more than 80% [18]. We employed statistical adjustments to estimate our sample as representative of the general population and calculate the proportion and odds ratios of being unvaccinated. In addition, the proportion of supporters falls within the range of other political party support polls, indicating that our results could be generalized to the Japanese population.

## Conclusions

Political beliefs may be associated with disparities in COVID-19 vaccination rates. In Japan, supporters of minor parties and non-partisans were more likely to remain unvaccinated compared to supporters of major parties. Investigating the underlying reasons for this disparity is essential for developing targeted strategies to overcome vaccination barriers.
